# Supplementary material for: The Immunometabolic Atlas: A tool for design and interpretation of metabolomics studies in immunology
Source: PLoS One. 2022 May 12;17(5):e0268408. doi: 10.1371/journal.pone.0268408 (PMC9098072; doi:10.1371/journal.pone.0268408)
Supplement: S1 Table — (DOCX) [file pone.0268408.s001.docx]

**S1 Table. Contents of IMA database.**

| **Type of data** | **Number included in the IMA** |
| --- | --- |
| Immune processes | 1 712 |
| Proteins | 3 101 |
| Metabolites | 97 525 |
| Immune process-protein associations | 69 641 |
| Protein-protein interactions | 411 286 |
| Protein-metabolite interactions | 172 291 |
| Metabolite-metabolite interactions | 664 |
