# Supplementary material for: The Immunometabolic Atlas: A tool for design and interpretation of metabolomics studies in immunology
Source: PLoS One. 2022 May 12;17(5):e0268408. doi: 10.1371/journal.pone.0268408 (PMC9098072; doi:10.1371/journal.pone.0268408)
Supplement: S2 Table — (DOCX) [file pone.0268408.s002.docx]

**S2 Table. Summary of superclass characteristics in the IMA**

| **Superclass** | **Number of metabolites** | **Number of proteins** | **Number of unique immune processes** | **Metabolite / protein ratio** | **Number of immune processes per protein** | **Number of unique immune processes per protein** |
| --- | --- | --- | --- | --- | --- | --- |
| Organic acids and derivatives | 714 | 138 | 386 | 5 | 57 | 3 |
| Organic nitrogen compounds | 134 | 29 | 148 | 5 | 74 | 5 |
| Organic oxygen compounds | 1278 | 162 | 431 | 8 | 84 | 3 |
| Benzenoids | 966 | 24 | 166 | 40 | 116 | 7 |
| Lipids and lipid-like molecules | 90309 | 164 | 467 | 551 | 95 | 3 |
| Nucleosides, nucleotides, and analogues | 152 | 436 | 639 | 0 | 78 | 1 |
| Organoheterocyclic compounds | 1786 | 72 | 291 | 25 | 52 | 4 |
| Phenylpropanoids and polyketides | 1915 | 9 | 74 | 213 | 62 | 8 |
| Alkaloids and derivatives | 113 | 1 | 16 | 113 | 16 | 16 |
| Organosulfur compounds | 174 | 9 | 77 | 19 | 42 | 9 |
